# An extremely rare case of an oversized accessory spleen: case report and review of the literature

**DOI:** 10.1186/s12893-019-0510-z

**Published:** 2019-04-27

**Authors:** V. Palumbo, M. Mannino, M. Teodoro, G. Menconi, E. Schembari, G. Corsale, I. Di Carlo, A. Toro

**Affiliations:** 1 Department of Surgical Sciences and Advanced Technologies “GF Ingrassia”, University of Catania, Cannizzaro Hospital, Via Messina 829, 95126 Catania, Italy; 2 Department of Radiology, Cannizzaro Hospital, Catania, Italy; 3 Department of General Surgery, E. Muscatello Hospital, Augusta, (SR) Italy

**Keywords:** Accessory spleen, Splenectomy, Trauma

## Abstract

**Background:**

The accessory spleen is a congenital defect characterized by a separated ectopic splenic parenchyma. The size is rarely more than 4 cm. The preoperative diagnosis is prohibitive preoperatively. The aims of the present manuscript were to present the case of a patient with a rare oversize accessory spleen and a review of the literature.

**Case presentation:**

A 15-year-old boy was admitted to the emergency department following blunt abdominal trauma.

The computed tomographic scan showed a traumatic rupture of the spleen and a 7-cm mass at the left side of the retroperitoneal space. Conservative treatment started and aborted after 4 h due to the onset of haemodynamic instability. Splenectomy was performed. An accessory spleen was discovered. A second large mass in the retroperitoneum was diagnosed as a second large accessory spleen that was also left in place. The postoperative course was uneventful, and the patient was discharged on the 7th postoperative day. Seven months later, the CT scan showed viability of both accessory spleens.

**Conclusion:**

An accessory spleen can be variously located and the retroperitoneal position is extremely uncommon. Preoperative diagnosis is still difficult, especially in emergency and as in our case, the literature shows the difficulty of reaching a diagnosis before surgery. The main misdiagnosis is neoplastic disease and for this reason accessory spleen can be wrongly removed.

An undiagnosed pre or intra operative retroperitoneal mass, closely to the spleen, have to be managed carefully. The diagnosis of accessory spleen needs to be ever considered as if found, represents a great possibility to conduct a normal life after splenectomy (of main spleen) for trauma.

## Background

The spleen appears approximately at the sixth week of embryologic life as a localized proliferation of the coelomic epithelium overlying the dorsal pancreatic endoderm. The proliferating cells invade the underlying angiogenic mesenchyme; as a result, it becomes condensed and vascularized in several adjoining areas that fuse together determining the formation of a lobulated spleen [1, 2]. Subsequently, the earlier lobulated feature of the spleen disappears, retaining notches on its upper surface, the remain unchanged in the adult [1].

The spleen is localized between the 9th and 11th left ribs in abdominal cavity, between the gastric bottom and the left hemidiaphragm. With a weight of approximately 200 g, it represents the largest lymphoid organ in the body [3]. It is fundamental for the haematological and immune system and is an important reserve of approximately 10–20% of the blood volume [4].

During its growth, the spleen can develop anomalies such as complete agenesis, multiple spleens or polysplenia, accessories spleens, isolated small additional splenunculi and persistent lobulation.

The accessory spleen is a congenital defect, with a separated ectopic splenic parenchyma due to an incomplete fusion of splenic masses during embryonic growth arising from the dorsal mesogastrium [2].

The aims of the present manuscript were to present a case of a patient with a rare oversized accessory spleen and a complete update of the literature concerning this rare condition.

## Case presentation

In June 2017, a 15-year-old boy was admitted to the emergency department following blunt abdominal trauma after being hit by a car while he was observing a car race.

The patient was intubated and was haemodynamically stable (blood pressure 100/60 mmHg, pulse rate 88/min); therefore, laboratory tests and CT-scan were performed.

**Fig. 1:**
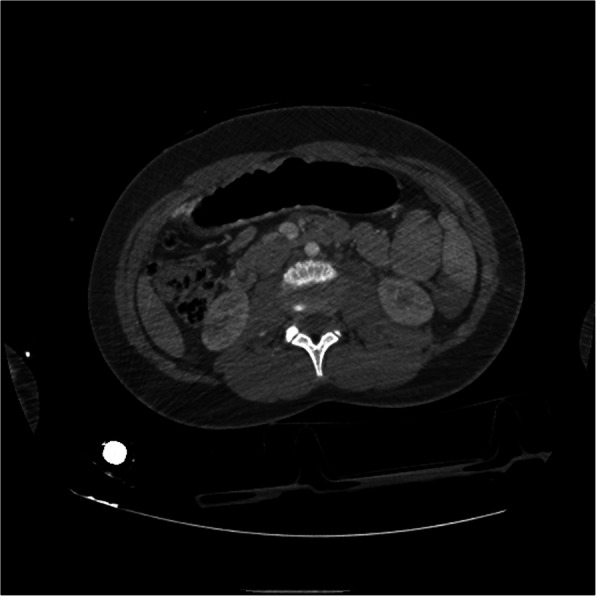
All spleens present in the patient. The injured one and the two accessories

The computed tomographic scan of the thorax and abdomen showed bilateral pleural effusions with rib fractures and a large haemoperitoneum associated with a traumatic rupture of the spleen with multiple injuries (grade III of the Organ Injury Scale, of AAST [5]) and a 7-cm mass at the left side of the retroperitoneal space (Fig. 1). A thoracic drain was inserted on the left side of the thorax, and non-operative management for the spleen started.

Haemoglobin decreased from 14.4 to 8.9 g/L during hospitalization with four hours of conservative treatment, with appearance of haemodynamic instability that was considered an indication for surgery.

**Fig. 2:**
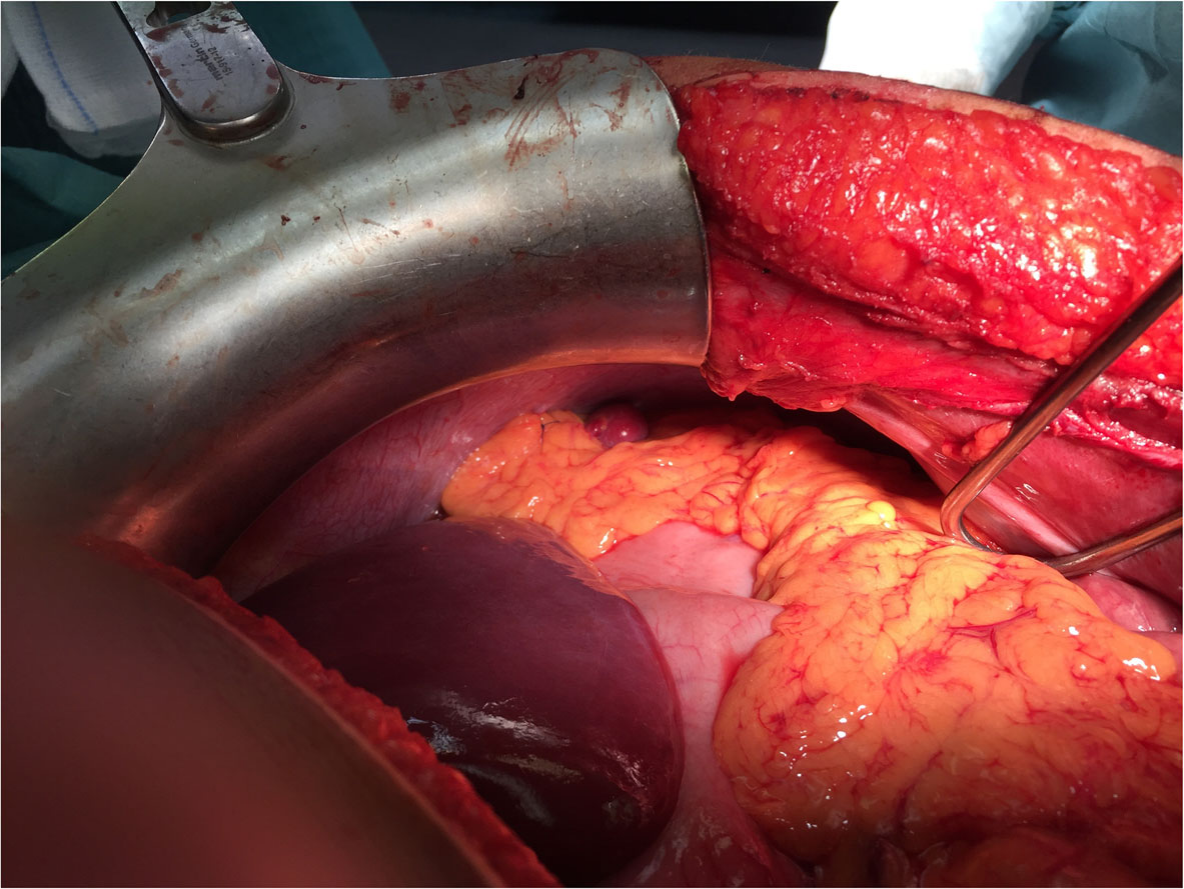
The small accessory spleen fixed in the native spleen position

An incision was made on the midline. The abdomen was packed and explored. The operation began with clearance of the haemoperitoneum. The spleen appeared with multiple longitudinal lesions in the visceral aspect. It was gently grasped and displaced medially towards the incision. The avascular peritoneal attachments and ligaments are incised with by electrocautery, followed by dissection of the splenogastric ligament and ligation of the short gastric vessels near the spleen to avoid injury or late necrosis of the gastric wall. The splenorenal, splenocolic and splenophrenic ligaments were divided. To avoid pancreatic injuries, dissection was carried out in close proximity to the hilum of the spleen, where the splenic artery and veins were identified, carefully dissected, doubly ligated and fixed with suture ligatures. After removal of the spleen, haemostasis was obtained and confirmed in a systematic fashion through careful inspection of the left subphrenic area, the greater curvature of the stomach and the short gastric vessel area, as well as the splenic hilum. Inspection of these areas showed an accessory spleen connected to the omentum by a vascular pedicle that was moved to the splenic fossa and fixed to the diaphragmatic peritoneum by prolene stitches to protect it from future traumatic injuries [6] (Fig. 2). A closed drainage was placed in the splenic fossa. Assuming that the 7-cm mass on the left side of the retroperitoneal space was an accessory spleen, given the vascular dynamics of the mass at the CT scan with contrast, it was decided to leave the retroperitoneal mass.

**Fig. 3:**
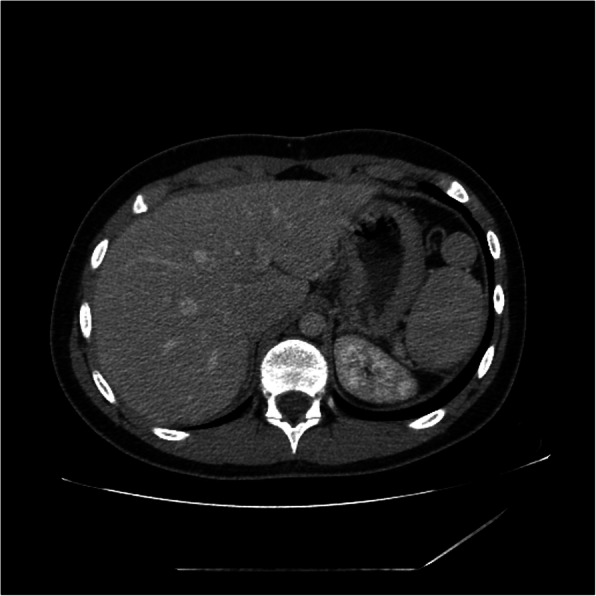
CT-scan 7 months after the surgical procedure showing the grown intraperitoneal accessory spleen in the splenic cavity and the retroperitoneal accessory spleen

The postoperative course was uneventful, and the patient was discharged on the 7th postoperative day. He was followed up for 7 months, during which he was well with no complications. A CT scan performed a couple of months after the surgical procedure showed the viability of the small spleen close to diaphragm. Radiological examination revealed the mass in the retroperitoneal space confirming a second accessory spleen (Fig. 3).

## Discussion and conclusions

The spleen is an important organ for the normal activity of the immune system. In fact, in case of asplenia, the patient is subject to an increased risk of sepsis or infections that may be caused by encapsulated bacteria such as *Neisseria meningitidis* or *Streptococcus pneumoniae* [7]. Furthermore, rare overwhelming post-splenectomy infection (OPSI) may occur, with fatal results, representing a risk in case of previous splenectomy.

Asplenia, polysplenia or multiple spleens, hyposplenia, accessory splenic nodule and persistent lobulation represent the congenital malformations of the spleen. Other rare congenital anomalies are reported in the literature, but they are not relevant to our study. By contrast, splenosis is an acquired alteration.

Asplenia is a very rare condition defined by the complete absence of the spleen. This condition is also known as Ivemark’s syndrome [8].

Polysplenia, or multiple spleens, is due to the absence of fusion of the primordial germs of the spleen. Therefore, between 1 and 6 small spleens are present in the abdominal cavity with dimensions between 1 cm and 6 cm; however, the total volume does not exceed the volume of a normal spleen [9]. Often this condition is associated with other malformations (viscero-atrial situs, cardiac anomalies, venous anomalies, pancreatic anomalies, intestinal malrotation, preduodenal portal vein) or malignancies [10].

Hyposplenia can be congenital, with a small spleen that can be the cause of immunodeficiencies in children [11]. Hyposplenia can be acquired due to sickle cell anaemia, celiac disease plasmodium malariae infection or alcoholic cirrhosis [12].

The lobulation of the spleen is an alteration of the spleen’s shape that has no pathological value [13].

Splenosis is defined as auto-transplantation of splenic tissue in a heterotopic location that primarily develops as a consequence of splenic trauma or surgery [14].

An accessory spleen is defined as ectopic splenic tissue that develops due to the failure of cell fusion during embryonic development while migrating from the midline to the left upper quadrant [15, 16]. They can be localized commonly next to the hilus and vascular pedicle, the tail of the pancreas, left ovary or left testis, in the greater omentum and in the mesentery of the small and the large intestine, along the greater curvature of the stomach and in the pouch of Douglas.

Accessory spleen can be present in 16% of the population, and they are found in 10–30% at autopsy [4]. Accessory spleens are usually single (85%); in some patients there are two (14%) and only in particular cases are found three or more (1%) [2, 17]. In this case, the total volume of all supernumerary spleens exceeded the normal volume of the only one natural spleen. Macroscopically, a typical accessory spleen usually appears as a solid mass, 1–2.5 cm in diameter. Masses larger than 4 cm are very rare (2–5) with a smooth, round, ovoid or minimally lobulated shape. Microscopically, it reproduces a splenic pattern. An accessory spleen commonly has a well-defined fibrotic capsule that separates the surrounding normal tissue [18].

These accessories spleens are often asymptomatic but may present as an abdominal mass connected to complications such as torsion, haemorrhage, spontaneous rupture or cyst formation. They are diagnosed during radiological examinations for other diseases. Their diagnosis becomes mandatory only in cases of haematological disorders [19]. In fact, some disease have to be cured by splenectomy and the remnant, if a very small spleen, can represent a problem requiring reintervention [20].

Accessory spleens look like normal spleens also in terms of immunologic functions. In haematologic disorders, when splenectomy is the treatment of choice, accessory spleens have to be located and removed because they will become hyperplastic and cause recurrence of the disease.

Accessories spleens may demonstrate compensatory hypertrophy and can grow to 3–5 cm after removal of the principal spleen [21].

Preoperative diagnosis of the accessory spleen is difficult, especially in emergencies. CT scans show a well-margined mass, similar to the splenic parenchyma on the contrast phase. Magnetic imaging can also be used to evaluate tissue aspects and the vascular pedicle of the accessory spleen. Only nuclear medicine imaging can confirm the diagnosis with scintigraphy performed with 99mTC-labelled colloids or TC-99 m heat damaged red blood cells because the colloid labelled with TC 99 m is taken from the reticulum-endothelium and makes visible the spleen, liver and bone marrow; however, it is necessary to suspect the diagnosis of accessory spleen for this procedure [22]. Therefore, often only surgical excision can safely confirm the diagnosis [23]. For these reasons, many surgical procedures have been done for the purpose of diagnosis.

**Fig. 4:**
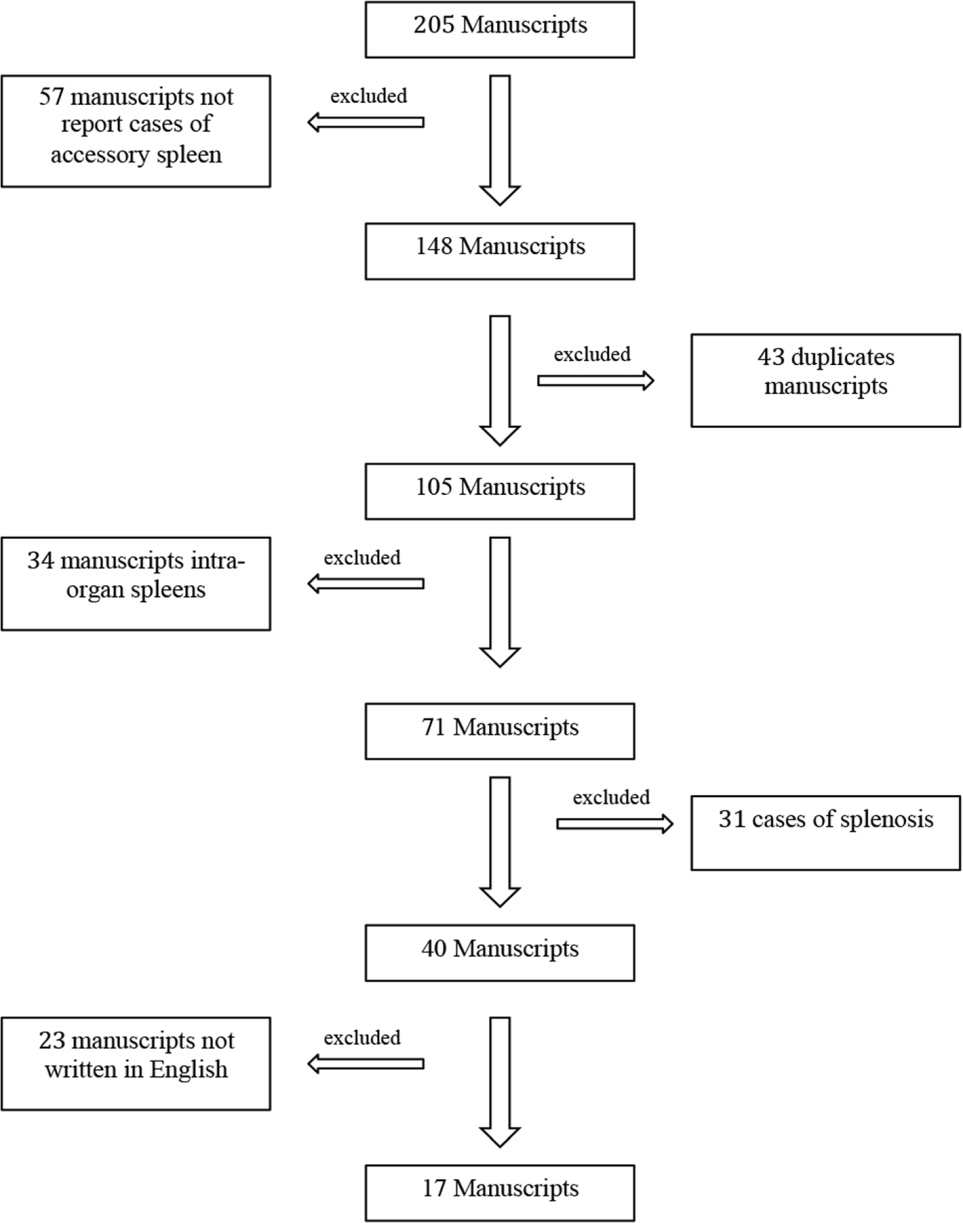
The flow chart of the Medline research

To perform the review of the literature, relevant articles in English were extensively searched from the MEDLINE (PubMed) database. The period of research was between 1999 and 2018. The date of the last search was July 10, 2018. The keywords used for the search were “spleen” “accessory spleen”, “retroperitoneal”, “retroperitoneal mass”, “retroperitoneal tumor”, “retroperitoneal spleen” “trauma”, “right”, and “omentum”. These words were used individually or with the Boolean operator “AND”. A total of 205 articles were analysed between 1999 at 2018. Of these 205 articles, 57 manuscripts (27, 8%) were excluded because they did not report cases of accessory spleen, 43 (21, 0%) were duplicates manuscripts, 34 manuscripts (16, 6%) analysed cases of intra-organ spleens, 31 articles (15, 1%) showed cases of splenosis and 23 manuscripts (11, 2%) were not written in English (Fig. 4). The remaining 17 manuscripts (8, 3%) reported 19 cases of accessory spleen (Table 1) [2, 15, 18, 20, 23–35] and were used for our manuscript.

**Table 1:** Review of literature of retroperitoneal accessory spleen

Year	Author	N°	Age	Sex	Side	Location	Dimension	Diagnosis
2018	Present case	1	15	M	Left	Retroperitoneal space	7 cm	Surgery
2017	Sbrana [24]	1	33	F	Left	Retroperitoneal space	7.7 × 6.0 × 5.8 cm	Surgery
2017	Xu [18]	1	65	M	Right	Parietal peritoneum	5 cm	Surgery
2017	Xia [25]	1	38	F	Left	Retroperitoneal space	4 × 2.5 cm	Surgery
2016	Maharaj [23]	1	44	M	Right	Retroperitoneal space	11 × 8 cm	Surgery
2015	Zhou [26]	1	40	F	Right	Retroperitoneal space	3,8 × 2.5 cm	Surgery
2015	Porwal [27]	1	50	M	Left	Suprarenal area	6 cm	Surgery
2014	Radu [2]	1	55	M	Left	Greater omentum	0.5/0.4 cm	Autopsy
		1	52	M	Left	Hilum spleen	0.5 cm	Autopsy
2013	Toutziaris [28]	1	58	F	Left	Retroperitoneal space	5.0 × 4.0 × 3,5 cm	Surgery
2013	Wu [29]	1	30	M	Left	Retroperitoneal space	3.3 cm	Surgery
2013	Arra [15]	1	24	M	Right	Right suprarenal region	20 cm	surgery
2011	Zhang [30]	1	22	F	Left	Greater omentum	12 × 38 cm	Surgery
2011	Tjaden [31]	1	69	M	Left	Retroperitoneal space	2 × 3 cm	Surgery
2008	Bergeron [20]	2	40	F	Left	Retropancreatici tissue	1 cm	Y probe
						Mesenteric of the spleen	1.4 cm	Surgery
2008	Kim [32]	1	68	M	Right	Retroperitoneal space	4 cm	Surgery
2006	Leon [33]	1	45	F	Left	Abdominal upper quadrant	6x6x4 cm	Surgery
2002	Budzynski [34]	1	45	F	Left	Retroperitoneal space	4 cm	Surgery
1999	Vural [35]	1	26	F	Left	Greater omentum	4.5 × 4.0 cm	Surgery

The analysed articles included only four cases of spleens larger than 7 cm, as in our case. This dimension is unusual because, according to the literature, these accessory spleens usually do not surpass 4 cm in diameter [18].

Diagnosis of these accessory spleens were possible preoperatively in only two cases. The first one was a patient affected by idiopathic thrombocytopenia who was operated on and found to have residual foci focused at a second procedure using a handled gamma probe [20]. The second case was a patient previously splenectomised for trauma that had a second trauma with an injured accessory spleen; in this case, the diagnosis was made on CT scan. Thirteen patients in our study underwent CT scan for diagnosis; in addition, other different diagnostic methods, including US [36], MRI [37] and PET [38] were used. One patient underwent only US, and another underwent only MRI. Finally, two cases were autopsies [2]. Of 15 cases, except for the two cases mentioned above, none obtained a diagnosis pre-operatively. The differential diagnoses ranged from adrenal gland tumour to fibroma, but all cases obtained the diagnosis of accessory spleen at surgery or after pathological examination. As in our case, the literature shows the difficulty of reaching a diagnosis before surgery. Our case was treated conservatively after diagnosis of an omental accessory spleen. At this moment, the radiologist was called to make a second examination of the CT scan; he confirmed the suspicion of a second accessory spleen. In four patients [24, 25, 29, 31], a previous splenectomy was done for various reasons, but this element did not help, and the diagnosis was not possible before surgery.

An accessory spleen can be variously located and the retroperitoneal position is extremely uncommon. Preoperative diagnosis is still difficult, especially in emergency and as in our case, the literature shows the difficulty of reaching a diagnosis before surgery. The main misdiagnosis is neoplastic disease and for this reason accessory spleen could be wrongly removed. For this reason undiagnosed pre or intra operative retroperitoneal mass, closely to the spleen, have to be managed carefully. The diagnosis of accessory spleen needs to be ever considered as if found, represents a great possibility to conduct a normal life after splenectomy (of main spleen) for trauma.

## Abbreviations

AAST: The American Association for the Surgery of Trauma
CT: Computed Tomography
MRI: Magnetic Resonance Imaging
OPSI: overwhelming post-splenectomy infection
PET: Positron Emission Tomography
US: Ultrasonography

## References

[1] Iorio F, Frantellizzi V, Drudi FM, Maghella F, Liberatore M (2015). Locally vascularized pelvic accessory spleen. J Ultrasound.

[2] Radu CC, Mutiu G, Pop O (2014). Accessory spleen. Romanian J Morphol Embryol.

[3] Impellizzeri P, Montalto AS, Borruto FA, Antonuccio P, Scalfari G, Arena F, Romeo C (2009). Accessory spleen torsion: rare cause of acute abdomen in children and review of literature. J Pediatr Surg.

[4] Unver Dogan N, Uysal II, Demirci S, Dogan KH, Kolcu G (2011). Accessory spleens at autopsy. Clin Anat.

[5] Moore EE, Shackford SR, Pachter HL, McAninch JW, Browner BD, Champion HR, Flint LM, Gennarelli TA, Malangoni MA, Ramenofsky ML (1989). Organ injury scal- ing: spleen, liver and kidney. J Trauma.

[6] Di Carlo I, Pulvirenti E, Toro A. New technique for spleen autotransplantation. Surg Innov. 2012;19:156–61. 10.1177/1553350611419867 21926100

[7] Di Carlo I, Primo S, Pulvirenti E, Toro A (2008). Should all splenectomised patients be vaccinated to avoid OPSI? Revisiting an old concept: an Italian retrospective monocentric study. Hepatogastroenterology..

[8] Varga I, Babala J, Kachlik D. Anatomic variations of the spleen: current state of terminology, classification, and embryological background. Surg Radiol Anat. 2018;40:21–9. 10.1007/s00276-017-1893-0 28631052

[9] Gayer g ZZ, Apter S, Atar E, Portnoy O, Itzchak Y (2001). CT findings in congenital anomalies of the spleen. Br J Radiol.

[10] Tawfik AM, Batouty NM, Zaky MM, Eladalany MA, Elmokadem AH (2013). Polysplenia syndrome: a review of the relationship with viscero-atrial situs and the spectrum of the extra-cardiac anomalies. Surg Radiol Anat.

[11] Scheuerman O, Bar-Sever Z, Hoffer V, Gilad O, Marcus N, Garty BZ (2014). Functional Hyposplenism is an important and underdiagnosed immunodeficiency condition in children. Acta Paediatr.

[12] Hommel B, Galloula A, Simon A, Buffet P (2013). Hyposplenism revealed by Plasmodium malariae infection. Malar J.

[13] Karakas HM, Tuncbilec N, Okten OO (2005). Splenic abnormalities: an overview on sectional images. Diagn Interv Radiol.

[14] Fremont RD, Rice TW (2007). Splenosis: a review. South Med J.

[15] Arra A, Ramdass MJ, Mohammed A, Okoye O, Thomas D, Barrow S (2013). Giant accessory right-sided suprarenal spleen in thalassaemia. Case Rep Pathol.

[16] Mortelé KJ, Mortelé B, Silverman SG (2004). CT features of the accessory spleen. AJR Am J Roentgenol.

[17] Kawamoto S, Johnson PT, Hall H, Cameron JL, Hruban RH, Fishman EK (2012). Intrapancreatic accessory spleen: CT appearance and differential diagnosis. Abdom Imaging.

[18] Xu SY, Sun K, Xie HY, Zhou L, Zheng SS, Wang W (2017). Accessory spleen located in the right parietal peritoneum: the first case report. Medicine (Baltimore).

[19] Orlando R, Lumachi F, Lirussi F (2005). Congenital anomalies of the spleen mimicking hematological disorders and solid tumors: a single-center experience of 2650 consecutive diagnostic laparoscopies. Anticancer Res.

[20] Bergeron E, Ratte S, Jeannotte S, Recoskie MJ (2008). The use of a handheld gamma probe fpr identifying two accessory spleens in difficult locations in the same patient. Ann Nucl Med.

[21] Beahrs JR, Stephens DH (1980). Enlarged accessory spleens: CT appearance in postsplenectomy patients. AJR Am J Roentgenol.

[22] Aggarwal R, Wagner T, Navalkissoor S (2013). Case report of Ct-99 sulfur single-photon emission computed tomography/computed tomography study differentiating tumor from accessory spleen. World J Nucl Med.

[23] Maharaj R, Ramcharan W, Maharaj P, Greaves W, Warner WA (2016). Right sided spleen laying retro-duodenal: a case report and review of the literature. Int J Surg Case Rep.

[24] Sbrana F, Zhou D, Zamfirova I, Leonardi N. Castleman’s disease: a rare presentation in a retroperitoneal accessory spleen, treated with a minimally invasive robotic approach. J Surg Care Report. 2017;(10):1–3. 10.1093/jscr/rjx195 PMC5633649 29026518

[25] Xia Z, Zhou Z, Shang Z, Ji Z, Yan W (2017). An unusual right-sided suprarenal accessory spleen misdiagnosed as an atypical Pheochromocytoma. Urology..

[26] Zhou JS, Hu HP, Chen YY, Yu JD (2015). Rare presentation of a right retroperitoneal accessory spleen: a case report. Oncol Lett.

[27] Porwall R, Singh A, Jain P (2015). Retroperitoneal accessry spleen presented as metastatic suprarenal tumor. A diagnotis dilemma. J Clin Diagn Res.

[28] Ch T, Kampantais S, Christopoulos P, Papaziogas B, Vakalopoulos I (2013). Compensatory enlargement of an accessory spleen mimicking a retroperitoneal tumor: a case report. Hippokratia..

[29] Wu ZS, Chiou SS, Lee JY, Chang YT (2013). Intraperitoneal accessory spleen and adrenal myelolipoma: removal by simultaneous bilateral posterior retroperitoneoscopy. Surg Laparosc Endosc Percutan Tech.

[30] Zhang C, Zhang XF (2011). Accessory spleen in the greater omentum. Am J Surg.

[31] Tjaden C, Werner J, Buechler MW, Hackert T (2011). Reactive hypertrophy of an accessory spleen mimicking tumour recurrence of metastatic renal cell carcinoma. Asian J Surg.

[32] Kim MK, Im CM, Oh SH, Kwon DD, Park K, Ryu SB (2008). Unusual presentation of right-side accessory spleen mimicking a retroperitoneal tumor. Int J Urol.

[33] Leon L, Labropoulos N, Hudlin CI, Macbeth AG, Matolo N, Andrus C (2006). Accessory spleen rupture in a patient with previous traumatic splenectomy. J Trauma.

[34] Budzynski A, Bobrzynski A, Sacha T, Skotnicki A (2002). Laparoscopic removal of retroperitoneal accessory spleen in patient with relapsing idiopatic thrombocytopenic purpura 30 years after classical splenectomy. Surg Endosc.

[35] Vural M, Kacar S, Kosar U, Altin L (1999). Symptomatic wandering accessory spleen in the pelvis:sonographic findings. J Clin Ultrasound.

[36] Görg C (2007). The forgotten organ: contrast enhanced sonography of the spleen. Eur J Radiol.

[37] Le D, Schierloh U, Van Nieuwenhuyse JP, Nchimi A (2016). Magnetic resonance imaging findings of Intrapancreatic accessory spleen. J Belg Soc Radiol.

[38] Bhure U, Metzger J, Keller FA, Zander A, Lago MP, Herring K, Strobel K (2015). Intrapancreatic accessory spleen mimicking neuroendocrine tumor on 68Ga-DOTATATE PET/CT. Clin Nucl Med.

